# Genotypic Variation in Drought-Season Stress Responses Among Traditional Fig (*Ficus carica* L.) Varieties from Mediterranean Transition Zones of Northern Morocco

**DOI:** 10.3390/plants14121879

**Published:** 2025-06-19

**Authors:** Mohammed Elmeknassia, Abdelali Boussakouran, Rachid Boulfia, Yahia Rharrabti

**Affiliations:** Laboratory of Natural Resources and Environment, Polydisciplinary Faculty of Taza, Sidi Mohamed Ben Abdellah University, Taza 35000, Morocco; mohammed.elmeknassia@usmba.ac.ma (M.E.); boussakouran@yahoo.fr (A.B.); rachid.boulfia@usmba.ac.ma (R.B.)

**Keywords:** Mediterranean climate adaptation, drought tolerance screening, osmotic stress markers, Moroccan traditional fig landraces

## Abstract

The fig (*Ficus carica* L.) is one of the oldest fruit crops cultivated in arid and semi-arid regions, valued for both its nutritional and economic importance; thus, ensuring sustainable fig production under climate change conditions is very important, as water scarcity increasingly affects fruit quality and production. Selecting and preserving resilient varieties among traditional varieties, representing centuries of local adaptation, is a vital strategy for addressing the challenges driven by climate change. In this context, this study assessed the physiological and biochemical parameters of the leaves of four fig landrace varieties (Fassi, Ghouddane, Nabout, and Ounq Hmam) grown in three different Mediterranean transitional zones of northern Morocco (Chefchaouen, Taounate, and Taza), during a single timepoint assessment conducted in late August 2023. The combined effects of location, variety, and their interactions on chlorophyll fluorescence (F_v_/F_m_), Soil Plant Analysis Development (SPAD) index, total chlorophyll content (ChlT), canopy temperature depression (CTD), proline content, protein content, total soluble sugar (TSS), hydrogen peroxide (H_2_O_2_), and malondialdehyde (MDA) were determined. Significant variation was observed among varieties and locations, with the location effect being observed for proline content, protein content, TSS, CTD, and ChlT, while variety had a stronger influence on SPAD, F_v_/F_m_, H_2_O_2_, and MDA. The results showed that Nabout and Ounq Hmam varieties had the greatest photosynthetic efficiency, as indicated by their elevated SPAD index, ChlT, and F_v_/F_m_ values, and showed lower sensitivity to oxidative stress (low proline content, H_2_O_2_, and MDA levels). In contrast, Ghouddane and Fassi displayed better stress tolerance, presenting higher levels of oxidative stress markers. Among locations, Chefchaouen showed the highest protein, TSS, H_2_O_2_, and MDA levels, reflecting active stress tolerance mechanisms. These variations were confirmed by principal component analysis, which revealed a clear separation between photosynthetically efficient varieties (Nabout and Ounq Hmam) and stress-tolerant varieties (Ghouddane and Fassi). More than a conventional crop physiology study, this work highlights the adaptive strategies in traditional Mediterranean fig germplasm that could be crucial for climate change adaptation. While our findings are limited to a single season, they offer valuable, practical insights that can inform grower decision-making in the near term, especially when considered alongside local knowledge and additional research.

## 1. Introduction

The fig (*Ficus carica* L.) is one of the oldest cultivated fruit crops, known for its ecological adaptability and its economic and nutritional importance [1,2]. Originally native to the Mediterranean region, fig trees expanded to warm temperate zones [3] due to their adaptability to diverse climates [4] and capacity to grow in various soil types [5,6]. Generally, warm temperate and arid Mediterranean regions seem to provide the best conditions for producing high-quality fig fruits [7,8,9,10]. According to the latest FAOSTAT data, figs were cultivated on 302,347 hectares of land worldwide, producing about 1,318,974 tons in 2023 [11]. Following these statistics, Turkey is the top producer (356,000 tons), followed by Egypt (193,057 tons), Morocco (119,166 tons), Algeria (116,183 tons), and Iran (73,483 tons). Indeed, Moroccan fig agriculture covers 69,737 hectares [11] and is concentrated in the northern regions of the country, including Taounate, Taza, Chefchaouen, Al Hoceima, Ouazzane, and Tetouane [12,13].

The fig tree represents a rich and varied phylogenetic heritage, reflected in its many varieties that are well adapted to different eco-geographical conditions and have good agronomic and ecological characteristics. As one of the world’s major fig producers, Morocco holds important fig genetic resources, including several international varieties [14,15,16,17]. A study conducted in the provinces of Taounate, Chefchaouen, Ouezzane, and El Jadida identified 43 local fig tree varieties [18]. Among these, the varieties recommended for fresh or dried autumn fig production include Nabout, Messari, Fassi, Ghouddane, Lassoune, Masfah, Hamri, and Ounq Hmam. In these regions, economic and sociocultural factors have contributed significantly to the preservation of traditional culture and the enhancement of genetic diversity [15,19]. Indeed, this genetic diversity within Moroccan fig germplasm, in the context of the Mediterranean basin, reflects a long history of domestication shaped by fig-specific selective pressures, including recurrent summer drought, shallow soils, and strong seasonal temperature fluctuations. These contrasting pressures have selected for distinct physiological and biochemical strategies, contributing to the rich intraspecific diversity observed across Moroccan fig populations [14,20]. In fact, fig trees have a significant ecological role to sustain the natural balance of agroecosystems in semi-arid and arid climates, stabilizing soil structure and promoting functional biodiversity. Often cultivated in association with other fruit trees such as olives, almonds, pomegranates, or grapevines, the fig tree displays a unique set of physiological adaptations that distinguish it within semi-arid and Mediterranean agroecosystems. These leaves are key components of a sophisticated water management system that has evolved over millennia of exposure to seasonal aridity. Their large, lobed, and thick structure enhances water retention and allows for precise regulation of transpiration [21,22]. The thick cuticle and a specialized distribution of trichomes reduce water loss and contribute to improved control over leaf water status [23,24]. In addition, their broad surface area creates localized microclimatic conditions that buffer leaf temperature and humidity [25]. Moreover, fig trees develop well-moistened root systems that facilitate nutrient uptake and promote excellent photosynthetic performance when soil water availability is unlimited [26,27]. However, despite their essential ecological role and unique physiological adaptations, native fig varieties in the Mediterranean area are currently exposed to serious genetic erosion caused by multiple biotic and abiotic stresses, including the expansion of monovarietal cropping systems and urban development.

Fig production and development are greatly influenced by geographical origin and associated factors, such as altitude, pedoclimatic conditions, and agricultural practices, including soil fertilization and irrigation [25,28,29]. Prolonged droughts, rising temperatures, and soil salinization are among the main effects of climate change, particularly in arid and semi-arid areas such as the Mediterranean. These changes disrupt plant phenological and physiological processes, including stomatal function, cell water status, and photosynthetic capacity [30,31]. As a result, environmental stresses significantly influence the physiology of fig trees, reducing their growth and productivity [32]. However, fig trees have developed diverse adaptive mechanisms and physiological processes to manage water scarcity [33,34]. These adaptive mechanisms include stabilizing the photosynthetic apparatus, mitigating oxidative damage through reactive oxygen species (ROS)-scavenging systems [35,36], and accumulating osmoregulatory substances such as soluble sugars and proline to maintain cellular homeostasis. Fig trees can increase photosynthetic rates through improved stomatal conductance in their leaves, thus allowing higher carbon dioxide (CO_2_) uptake and assimilation [36,37]. Indeed, recent studies have shown that genotype [33], leaf and fruit development stages [9,38], growing region, and edaphoclimatic conditions [34] all influence gas exchange capacity. Moreover, fig trees accumulate osmoprotectors (proline, soluble sugars, and antioxidants) and improve their antioxidant defenses to minimize oxidative stress produced by reactive oxygen species [39]. Under drought conditions, figs also show changes in chlorophyll fluorescence (F_v_/F_m_), chlorophyll index, and leaf number [34]. In this regard, chlorophyll content and chlorophyll fluorescence have been widely used to evaluate the adaptation of the photosynthetic machinery and plant performance under stressed environments [40]. Furthermore, stress-related biochemical markers (proline, soluble sugars, and antioxidants) can provide a global comprehension of stress tolerance mechanisms in fig trees [41].

The northern Moroccan regions, particularly Chefchaouen, Taounate, and Taza, present diverse agroclimatic conditions, with considerable variations in temperature, rainfall, and soil characteristics. These environmental differences influence the physiological performance of fig trees, particularly during the peak stress period for Mediterranean figs that coincides with late August. During this time, fig trees deal with maximum water deficit, high temperatures, and the physiological demands of fruit maturation. However, landrace varieties such as Fassi, Ghouddane, Nabout, and Ounq Hmam each carry unique adaptive genetic packages that could be crucial for climate change resilience. While previous studies have characterized individual fig varieties under controlled conditions, no research has systematically compared the physiological responses of traditional Moroccan landraces across natural environmental gradients during peak summer stress periods. Therefore, this study aimed to identify potential drought-tolerant fig germplasm from three locations in northern Morocco and to discern their physiological attributes to seasonal drought stress.

## 2. Results

### 2.1. Analyses of Variance

Mean squares of the combined analyses of variance for the studied physiological and biochemical traits of fig leaves are summarized in Table 1. The results showed that variety, location, and interaction significantly affected all the study parameters. Location effect significantly influenced protein content and TSS, accounting for 44% and 75% of total variance, respectively. A nearly equal influence of location and variety was observed for ChlT, CTD, and proline, with variation percentages ranging between 38% and 48%. In contrast, the variety effect was mainly exhibited for SPAD (56%), F_V_/F_m_ (67%), H_2_O_2_ (89%), and MDA (94%). Location by variety interaction significantly affected SPAD (27%) and protein content (41%), while its effect on the remaining traits was of relatively minor magnitude.

### 2.2. Effect of Location and Variety

**Figure 1:**
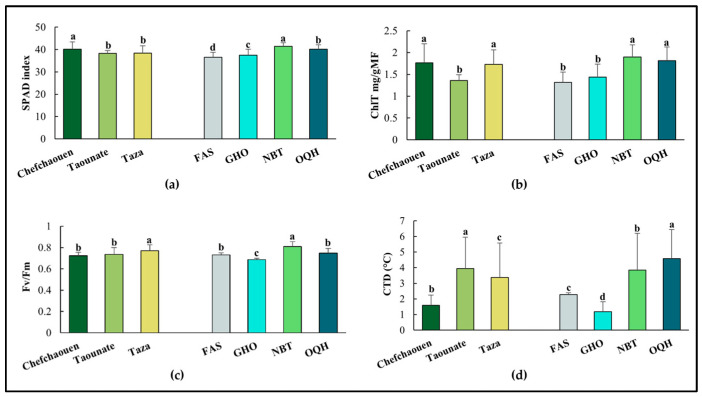
Mean values of SPAD index (**a**), total chlorophylls, ChlT (**b**), chlorophyll fluorescence, F_v_/F_m_ (**c**), and canopy temperature depression, CTD (**d**) of four fig varieties (FAS: Fassi; GHO: Ghouddane; NBT: Nabout; OQH: Ounq Hmam) grown at three different locations (Chefchaouen, Taounate, and Taza). Bars followed by the same letter are not significantly different according to Duncan test (*p* < 0.05).

The mean values of SPAD index, ChlT, F_v_/F_m_, and CTD are presented in Figure 1. Fig leaves from Chefchaouen had higher values of SPAD index (40.113) and ChlT (1.764 mg/g FW), and the lowest values of F_v_/F_m_ (0.722) and CTD (1.596 °C). Taounate exhibited the best scores for SPAD index and ChlT (38.213 and 1.356 mg/g FW, respectively). The highest value of F_v_/F_m_ (0.77) was observed in Taza. Among varieties, significant differences were observed, with Nabout showing the highest levels of most parameters (SPAD index = 41.423, ChlT = 1.9 mg/g FW, and F_v_/F_m_ = 0.809) except for CTD, where Ounq Hmam had higher values (4.581 °C). Ghouddane was characterized by the lowest value of F_v_/F_m_ and CTD (0.685 and 1.187 °C, respectively), whereas the lowest value of SPAD (36.506) index was observed in Fassi.

**Figure 2:**
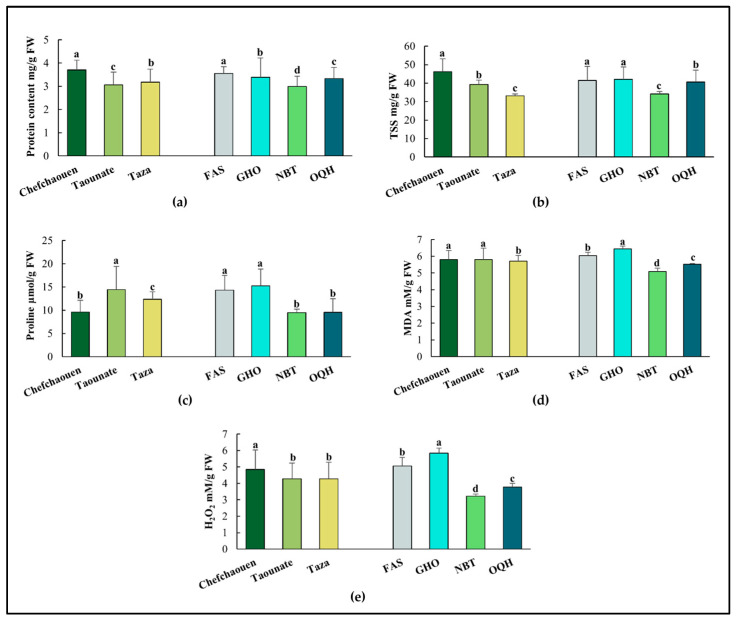
Mean values of protein content (**a**), total soluble sugars, TSS (**b**), proline (**c**), malondialdehyde, MDA (**d**), and hydrogen peroxide, H_2_O_2_ (**e**) of four fig varieties (FAS: Fassi; GHO: Ghouddane; NBT: Nabout; OQH: Ounq Hmam) grown at three different locations in northern Morocco (Chefchaouen, Taounate, and Taza). Bars followed by the same letter are not significantly different according to Duncan test (*p* < 0.05).

Significant variations were observed for biochemical parameters such as proline content, protein content, TSS, H_2_O_2_, and MDA (Figure 2). In fact, Chefchaouen presented the highest values of protein content, TSS, H_2_O_2_, and MDA (3.705 mg/g FW, 46.18 µmol/g FW, 4.851 mM/g FW, and 5.805 mM/g FW, respectively); however, Taounate recorded higher values of proline content (4.992 µmol/g FW). Among varieties, Ghouddane recorded the highest values of proline content, TSS, H_2_O_2_, and MDA (15.246 µmol/g FW, 42.015 mg/g FW, 5.837 mM/g FW, and 6.443 mM/g FW, respectively), followed by Fassi. However, leaves in Nabout had the lowest concentrations of proline content, TSS, protein content, H_2_O_2_, and MDA (9.465 µmol/g FW, 34.078 mg/g FW, 2.992 mg/g FW, 3.219 mM/g FW, and 5.078 mM/g FW, respectively).

### 2.3. Relationships Between Parameters

Table 2 presents the correlation matrix of the studied physiological and biochemical traits of fig leaves. Positive correlations were found between F_v_/F_m_, SPAD, and ChlT, while all these variables were negatively correlated with H_2_O_2_, MDA, and proline content. In fact, the highest and most significant relationship (r = 0.834 ***) was observed between SPAD and ChlT. CTD was positively associated with F_v_/F_m_ (r = 0.652 ***) and negatively associated with H_2_O_2_ (r = −0.633 ***) and MDA (r = −0.509 ***). Protein content was positively significantly correlated with TSS (r = 0.556 ***), H_2_O_2_ (r = 0.351 *), and MDA (r = 0.351 *). Additionally, TSS showed a negative association with F_v_/F_m_ (r = −0.499***), but it was positively correlated with H_2_O_2_ (r = 0.580 ***) and MDA (r = 0.488 ***). Moreover, positive correlations were found between proline content and both H_2_O_2_ (r = 0.492 ***) and MDA (r = 0.683 ***). Finally, H_2_O_2_ was positively correlated with MDA (r = 0.930 ***).

### 2.4. Principal Component Analysis

**Figure 3:**
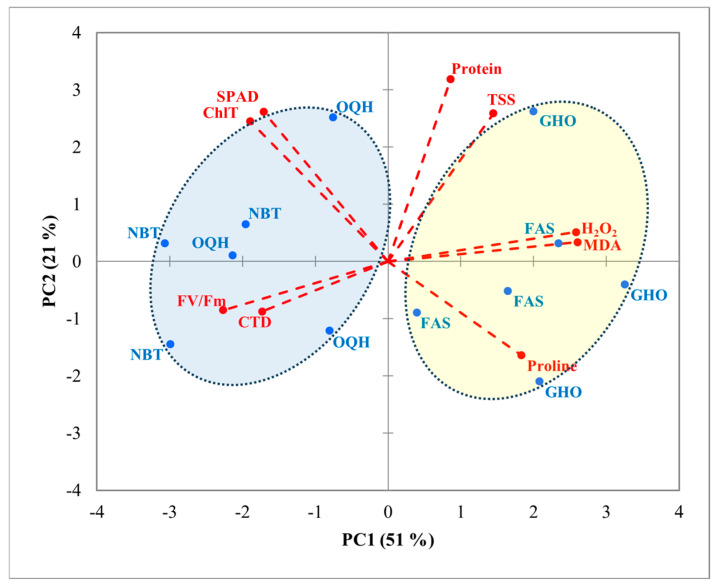
PCA projections on axes 1 and 2, accounting for 72% of the total variance. Eigenvalues of the correlation matrix are symbolized as vectors representing traits that most influence each axis. The 12 points representing trait means for each variety (FAS: Fassi, GHO: Ghouddane, NBT: Nabout, OQH: Ounq Hmam) are plotted on the plane determined by axes 1 and 2. F_v_/F_m_: chlorophyll fluorescence, ChlT: total chlorophyll content, TSS: total soluble sugars, CTD: canopy temperature depression, MDA: malondialdehyde, H_2_O_2_: hydrogen peroxide.

**Figure 4:**
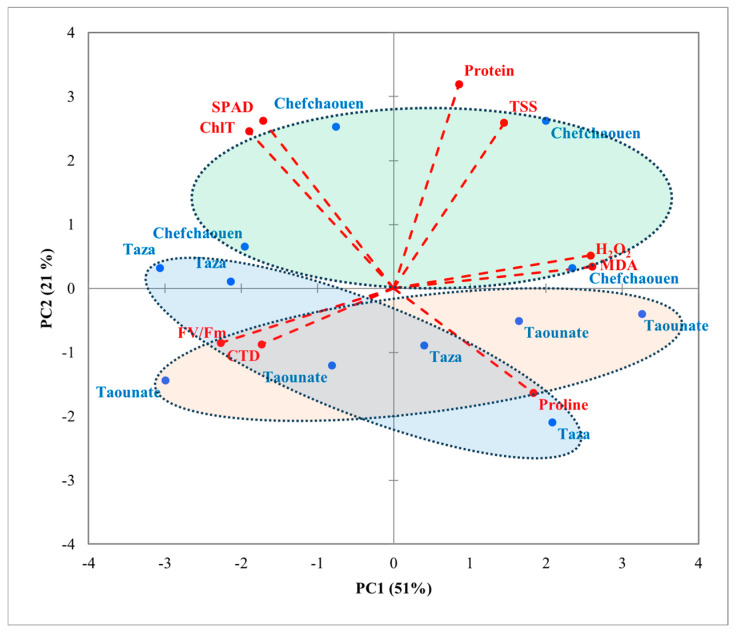
PCA projections on axes 1 and 2, accounting for 72% of the total variance. Eigenvalues of the correlation matrix are symbolized as vectors representing traits that most influence each axis. The 12 points representing trait means for each location (Chefchaouen, Taounate, and Taza) are plotted on the plane determined by axes 1 and 2. F_v_/F_m_: chlorophyll fluorescence, ChlT: total chlorophyll content, TSS: total soluble sugars, CTD: canopy temperature depression, MDA: malondialdehyde, H_2_O_2_: hydrogen peroxide.

PCA was conducted on the correlation matrix, using mean values to determine relationships between the studied factors and various parameters. Results showed that the first two PC axes explained about 72% of the total variance, with PC1 accounting for 51% and PC2 for 21% (Figure 3 and Figure 4). PC1 clearly separated protein content, TSS, H_2_O_2_, MDA, and proline content in the positive direction from SPAD, ChlT, F_v_/F_m_, and CTD in the opposite direction. The second axis (PC2) allowed discrimination between Chlt, SPAD, protein content, and TSS towards the upper side and both F_v_/F_m_, CTD, and Proline content downwards. Regarding varieties (Figure 3), a clear distinction among the four varieties was shown along PC1. Ghouddane and Fassi were marked by higher values of proline content, H_2_O_2_, and MDA on the right side, while Nabout and Ounq Hmam were correlated with SPAD, ChlT, F_v_/F_m_, and CTD on the opposite side. A separation of locations was detected along PC2 (Figure 4). Chefchaouen was positioned towards the positive side, with higher values of ChlT, SPAD, protein content, and TSS, while Taounate, in the opposite direction, interacted with proline content, F_v_/F_m_, and CTD. Points associated with the Taza location overlapped between Chefchaouen and Taounate.

## 3. Discussion

For a sustainable increase in fig production under climate change conditions, it is necessary to select new fig plants while considering productivity, drought tolerance, and cultural requirements. Local fig varieties are the fruit of a lengthy adaptation to climate and soil conditions in Morocco [14]. However, there are no previous studies on the physiological and biochemical behavior of local varieties based on the combined effects of location and variety and their interactions. Hence, this work focused on the study of four local fig varieties grown at three different locations in northern Morocco.

The fig plant is characterized by larger and thicker leaves, which enable enhanced chlorophyll production and facilitate efficient gas exchange, thereby conferring a robust adaptation to the arid Mediterranean climate [42]. Indeed, climatic factors such as higher temperature, low rainfall, and low light negatively affect leaf chlorophyll content and photosynthetic parameters, including net photosynthetic assimilation, stomatal conductance, and transpiration rate in fig trees [33,43].

The results of this work indicate that varietal and environmental factors strongly affect the physiological and biochemical parameters of fig leaves. This finding aligns with previous research showing that genotype plays a dominant role in determining photosynthetic performance and antioxidant capacity in fig varieties, while environmental conditions, such as water availability, temperature, and soil characteristics, modulate primary metabolism, including protein content and TSS [7,33,43,44].

Our results revealed that Chefchaouen leaves had the highest SPAD index and ChlT values, suggesting optimal environmental conditions for chlorophyll synthesis. This was confirmed by the high positive correlation between ChlT and the SPAD index. Taounate also showed strong scores for these two parameters, reinforcing its suitability for maintaining robust photosynthetic activity. In fact, the SPAD index and ChlT are crucial indicators of photosynthetic capacity and overall plant health [35,45,46]. These results align with research emphasizing the importance of environmental adaptation in photosynthetic performance [44,47]. Among varieties, Nabout displayed the highest SPAD index and ChlT, reflecting its genetic predisposition for superior chlorophyll production. These findings coincide with those of Maatallah et al. [7], who reported that Mlouki and Assal cultivars grown under warm climate conditions of Tunisia had the greatest amounts of chlorophyll and net photosynthetic assimilation.

The maximum photochemical efficiency (F_v_/F_m_) is a reliable indicator of photosynthetic apparatus functionality and light-dependent reactions, including water splitting, electron transport, establishment of the pH gradient across the thylakoid membrane, and ATP synthesis [48,49]. In fact, F_v_/F_m_ represents an estimator of the maximal photochemical efficiency of PSII and is often misinterpreted as a specific indicator of PSII photoinhibition due to the damage of PSII reaction centers [50]. Our results showed that the F_v_/F_m_ ratio varied between 0.69 and 0.81 in all varieties in accordance with data from Mardinata et al. [35]. The maximum F_v_/F_m_ ratio was recorded in Nabout (0.81), indicating its ability to maintain efficient photochemistry under variable environmental conditions. This was confirmed by the high positive correlation between F_v_/F_m_ and the SPAD index. In contrast, Ghouddane was characterized by the lowest F_v_/F_m_ (0.685), which may at first suggest stress-induced damage. However, this was most likely an attempt at photoprotection by means of inactivation of PSII reaction centers. F_v_/F_m_ represents a quantum yield of PSII that will be low not only when the PSII is inactivated but also due to thermal dissipation through slow-relaxing nonphotochemical quenching (NPQ) [50,51]. This intricate mechanism safeguards the photosynthetic machinery from light-induced damage, ensuring optimal photosynthetic efficiency [52,53]. Indeed, traditional drought-adapted varieties often show constitutively lower F_v_/F_m_ values as a protective mechanism against photoinhibition [51,54,55]. This landrace variety might be downregulating photochemical efficiency to avoid overexcitation and limit ROS generation, especially under conditions of reduced stomatal conductance [33]. Likewise, the low F_v_/F_m_ ratio (0.722) observed in Chefchaouen reflects an adaptive response where PSII is selectively protected by non-photochemical quenching and antioxidant activity processes enhanced during midday or seasonal drought [33,36,49].

Plants produce and accumulate osmolytes under abiotic stresses, including amino acids (proline), soluble sugars, and proteins, for maintaining cell turgor [56]. Furthermore, accumulation of such compounds would produce higher negative water potential in plants, a condition required for absorbing and holding water in plant cells [57]. Our results demonstrated that Chefchaouen exhibited the highest protein content and total soluble sugars, indicating enhanced metabolic reprogramming and osmoprotective adjustments as core mechanisms of stress tolerance in this environment. Valluru and Van den Ende [58] showed the important role of soluble sugars in osmotic adjustment and cell protection by inducing direct detoxification processes of reactive oxygen species or by indirectly stimulating the antioxidative defense system. Additionally, accumulation of organic solutes such as soluble carbohydrates and proline to maintain cell turgor was observed in drought-stressed olive and wheat leaves [59,60]. Regarding varieties, Ghouddane recorded the highest proline content, indicating a strong drought tolerance mechanism. For traditional Mediterranean fig varieties, constitutive proline accumulation may be a genetic adaptation to osmotic adjustment [36,39]. This idea is strongly reinforced by the negative correlation between F_v_/F_m_ and proline, suggesting that traditional varieties with strong constitutive osmotic defenses (high proline) may adopt a conservative photochemical strategy (lower F_v_/F_m_). In fact, proline accumulation is known to be a key response to osmotic stress, acting as an osmoprotectant to stabilize cellular structures [39,61]. In addition to osmotic regulation, proline contributes to drought tolerance by (i) mimicking water molecules to protect cellular structures from damage [62], (ii) stabilizing redox balance within cells [63], and (iii) serving as a reservoir of carbon, nitrogen, and reducing equivalents essential for plant recovery following drought stress [64]. On the other hand, Nabout exhibited the lowest concentrations of proline content, TSS, and protein content, indicative of a stress-avoidance strategy rather than large-scale osmotic adjustment. However, its superior SPAD index and F_v_/F_m_ values reflect robust PSII performance and chlorophyll stability under moderate water deficit. This pattern aligns with recent findings in *Ficus carica* genotypes, where minimal osmolyte accumulation correlated with sustained PSII performance and efficient water use [43,44].

H_2_O_2_ and MDA are well-known plant stress indicators. MDA results from lipid peroxidation of polyunsaturated fatty acids [65,66], whereas H_2_O_2_ is a central reactive oxygen species produced in organelles during environmental stress [67,68]. They are often interpreted as signals of damage, but in traditional Mediterranean crops, they frequently underpin active stress-tolerance mechanisms. In salt-stressed rosemary, transient MDA accumulation coincides with a surge in antioxidant enzyme activity, suggesting a protective rather than purely deleterious role [69]. Similarly, H_2_O_2_ generated in chloroplasts functions as a key retrograde signaling molecule during environmental stress, linking chloroplast redox changes to nuclear gene regulation [70,71,72]. Recent reviews further emphasize that controlled ROS production primes defense pathways, enhancing osmoprotectant synthesis and stabilizing photosystems under abiotic stresses like drought and heat [73,74]. In our study, the highest H_2_O_2_ and MDA levels recorded at Chefchaouen and expressed by Ghouddane could reflect a primed antioxidative network, comprising increased ROS scavenging enzymes and osmoprotectant synthesis. This interpretation is further supported by the negative correlation between F_v_/F_m_ and oxidative markers, suggesting a protective downregulation of photochemical efficiency under stress, a known adaptive trait in drought-tolerant varieties. In contrast, Nabout maintained comparatively low H_2_O_2_ and MDA levels, which correlated with its high F_v_/F_m_ and SPAD values, reflecting an ability to sustain photochemical performance with minimal engagement of high-amplitude oxidative defenses, highlighting a distinct adaptive mechanism from the ROS-primed tolerance seen in Ghouddane. This profile is characteristic of a stress-avoidance strategy, where ROS production is minimized through efficient stomatal regulation and non-photochemical quenching rather than by relying on ROS-mediated signaling cascades. Such varieties exhibit constitutive antioxidant enzyme activities, notably superoxide dismutase and ascorbate peroxidase, that prevent excessive ROS accumulation under peak stress [43,47]. PCA plots revealed a clear separation among the four fig varieties. Ghouddane and Fassi clustered on the right side of PC1, associated with higher levels of proline content, H_2_O_2_, and MDA. This clustering suggests that these varieties exhibit stronger stress tolerance, which may be beneficial for their survival under harsher environments. These findings align with research by Choudhury et al. [73], who noted that elevated levels of reactive ROS and proline content are common indicators of plants coping with oxidative stress. In contrast, Nabout and Ounq Hmam clustered on the left side of PC1, associated with higher photosynthetic efficiency indicators, such as SPAD index, ChlT, and F_v_/F_m_. These varieties appear to be more suited to environments where photosynthesis can be prioritized over inducible stress responses. Recent work by Del Rosario Jacobo-Salcedo et al. demonstrated that fig genotypes with this profile maintain higher photosynthetic rates and water-use efficiency through optimized stomatal conductance and cyclic electron flow [44]. Additionally, the PCA plot revealed a clear separation of the three locations. PC2 reflects a trade-off between investment in growth-related metabolism (positive side) and deployment of drought-avoidance mechanisms (negative side). Chefchaouen, clustered on the positive side of PC2, combined high ChlT, SPAD, protein, and TSS levels, suggesting resource-rich conditions that support robust primary metabolism and carbon assimilation. These values likely reflect Chefchaouen’s higher rainfall, cooler microclimate, and moisture-retentive soils, which together promote nitrogen uptake, chlorophyll synthesis, and carbohydrate accumulation. In contrast, Taounate clustered on the negative side of PC2, characterized by elevated proline content, F_v_/F_m_, and CTD, indicating a stress-avoidance strategy under drier, more thermally demanding conditions [49,73].

## 4. Materials and Methods

### 4.1. Plant Sampling and Site Description

**Figure 5:**
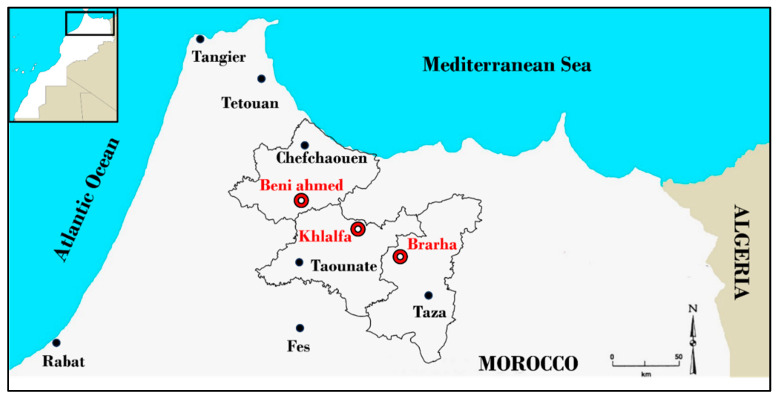
Geographical localization of the three study sites.

Four Moroccan fig varieties (Fassi: FAS, Ghouddane: GHO, Nabout: NBT, and Ounq Hmam: OQH) were chosen from the most cultivated Moroccan figs. Each variety was marked in triplicate at three different locations in northern Morocco: Chefchaouen (Beni Ahmed, 34°50′23.7″ N 5°04′42.9″ W), Taounate (Khlalfa, 34°39′56.4″ N 4°37′01.6″ W), and Taza (Brarha, 34°28′08.8″ N 4°20′42.4″ W) (Figure 5). These locations were chosen for their extensive fig cultivation and commercial production. They exhibit different agroclimatic and edaphic characteristics that define a specific environment for fig cultivation. Taza, situated at 620 m altitude, is characterized by the hottest summer temperatures (up to 30 °C in August) and the highest vapor pressure deficit (2.55 kPa in August), coupled with low annual rainfall (433 mm) and sandy–loam soils with poor water-holding capacity, making it the most arid and drought-prone environment. Taounate, located at 580 m altitude, has slightly less heat stress (maximum 29.8 °C in August) and moderate vapor pressure deficit (2.47 kPa in August), receiving 477 mm of annual rainfall. Its silty soils offer moderate water retention and drainage capacity, placing it in an intermediate stress zone. Finally, Chefchaouen, at 510 m altitude and under coastal influence, has the most buffered microclimate, with cooler summer temperatures (maximum 29.6 °C in August), the lowest vapor pressure deficit (1.83 kPa in August), and the highest annual precipitation (624 mm). Its clay–loam soils, with moderate permeability and high hydraulic retention, contribute to greater soil moisture availability, reducing drought intensity. These gradients in elevation, temperature, atmospheric dryness, precipitation, and soil water dynamics establish Taza as the most stressful location, Chefchaouen as the most favorable, and Taounate as ecologically intermediate. The study was carried out in late August 2023, which corresponds to the peak stress period for fig trees in the Mediterranean region, to highlight genotypic differences in long-term stress acclimation. Across the three sites, orchards were managed under rainfed conditions with uniform agronomic practices, and no biostimulants were applied during the study period. For SPAD, F_v_/F_m_, and CTD measurements, four healthy, fully expanded, and sun-exposed leaves were randomly selected from current-year shoots of the four cardinal sides of each tree to ensure representative light exposure and minimize positional bias within the canopy. Those measurements were carried out in three well-developed, uniformly aged fig trees for each variety. At least five recordings were taken per leaf to ensure data reliability and account for intra-leaf variability. Once the in situ measurements were completed, selected leaves were collected, immediately placed in an ice box, and transported to the Laboratory of Natural Resources and Environment at the Polydisciplinary Faculty of Taza, where they were stored at −20 °C until biochemical trait and total chlorophyll (ChlT) analyses were performed.

### 4.2. Physiological Trait Determination

#### 4.2.1. Chlorophyll Fluorescence (F_v_/F_m_)

Chlorophyll fluorescence was recorded between 12:00 and 13:00 using a portable fluorometer (OS-30p, Opti-Science Inc., Hudson, NH, USA) following the procedure reported by [59]. Intact fig leaves were dark-adapted for 20 min using light-exclusion clips to determine the minimum fluorescence (F_0_). Then, the maximum fluorescence in light (F_m_) was determined after applying a saturating actinic light pulse (3000 μmol m^−2^ s^−1^). These values were used to calculate the maximum quantum efficiency of photosystem II (PSII) as F_v_/F_m_ = (F_m_ − F_0_)/F_m_. All measurements were taken under stable, cloud-free conditions.

#### 4.2.2. SPAD Index

SPAD index was determined using a handheld SPAD 502 Plus chlorophyll meter (Konica Minolta, Osaka, Japan), which measures the difference in transmittance between red (650 nm) and infrared (940 nm) wavelengths through the leaf, yielding a three-digit SPAD value [75]. Measurements were performed on at least four fully expanded, sun-exposed leaves per variety.

#### 4.2.3. Total Chlorophyll Content (ChlT)

Chlorophyll content was determined following the method described by Wellburn [76], using 20 mg of finely cut leaf discs incubated in 7 mL of dimethyl sulfoxide (DMSO) at 65 °C for 1 h. Given the thick cuticle and high structural heterogeneity of fig leaves, samples were taken from the interveinal lamina tissue to avoid major veins and to improve representativeness. To minimize chlorophyll degradation and pheophytin formation, all extractions were carried out under dim light and in the presence of a small quantity of calcium carbonate (CaCO_3_). Absorbance was recorded at 663 nm and 645 nm using a spectrophotometer (JENWAY 6100, Dunmow, Essex, UK), and the total chlorophyll content was calculated using standard equations.

#### 4.2.4. Canopy Temperature Depression (CTD)

CTD was calculated as the difference between ambient air temperature and leaf surface temperature, with positive values indicating that leaves were cooler than the surrounding air. Leaf temperature was recorded using a handheld infrared thermometer (Model 320-EN-00, BENETECH, China) by targeting the adaxial surface of fully exposed leaves at a 45° angle to minimize reflectance artifacts. Measurements were performed between 11:00 and 12:00 under clear-sky, sunny conditions with minimal wind. Air temperature was recorded simultaneously using a shaded digital thermometer [60].

### 4.3. Biochemical Trait Determination

#### 4.3.1. Proline Content

Free proline content was determined using the colorimetric method adapted from Bates et al. [77]. Fresh fig leaf tissue (200 mg) was homogenized in 4 mL of 3% sulfosalicylic acid and centrifuged at 1000 rpm for 10 min. Two milliliters of the supernatant were mixed with 2 mL of ninhydrin reagent (2.5% in a mixture of 60% acetic acid and 2.5 M phosphoric acid) and 2 mL of 100% acetic acid (1:1:1, *v*/*v*/*v*), then incubated at 100 °C for 60 min. After cooling, the reaction mixture was extracted with 3 mL of toluene, and the absorbance of the colored upper phase was measured at 520 nm. Free proline content was calculated using a standard curve prepared with L-proline.

#### 4.3.2. Total Soluble Sugars (TSS)

Total soluble sugars (reported as mg·g^−1^ FW) was estimated according to the technique of Dubois et al. [78]. Leaf sections (250 mg) were ground in 5 mL of 80% methanol (*v/v*) and boiled at 90 °C for 30 min. Once cooled, 1 mL of the extract was combined with 1 mL of phenol (5%) and 5 mL of concentrated sulfuric acid. After agitation and cooling of the reagent mixture, absorbance was read at 490 nm using methanol as a blank. Total soluble sugar concentration was estimated using glucose solution as a standard curve.

#### 4.3.3. Hydrogen Peroxide (H_2_O_2_)

Hydrogen peroxide content was measured using the protocol described by Sarker and Oba [79]. Leaf sample (250 mg) was homogenized with 5 mL of trichloroacetic acid (0.1%) and centrifuged at 10,000× *g* for 5 min. Then, the supernatant (0.2 mL) was mixed with 1 mL of KI (1 M) and 0.8 mL of phosphate buffer (pH 7), and the absorbance of this mixture was read at 390 nm (Spectrophotometer JENWAY 6100, Dunmow, Essex, UK). The hydrogen peroxide content was measured using a standard curve of H_2_O_2_.

#### 4.3.4. Malondialdehyde (MDA)

MDA content was measured to evaluate lipid peroxidation. It was estimated using the method described by Sarker and Oba [79]. Frozen samples (250 mg) were mixed with 5 mL of trichloroacetic acid (0.1%) in an ice bath, and the solution was centrifuged at 10,000× *g* for 10 min. Then, 1 mL of the obtained supernatant was added to 4 mL of the prepared mixture of trichloroacetic acid (TCA, 20%) and thiobarbituric acid (TBA, 0.5%), and was subsequently heated at 95 °C for 10 min. After centrifugation (10,000 rpm/5 min), the absorbance was measured at 532 nm (Spectrophotometer JENWAY 6100, Dunmow, Essex, UK) in order to determine the MDA content (mmol/g FW).

#### 4.3.5. Protein Content

Protein content was determined following the method described by Sarker et al. [80]. For protein extraction, 200 mg of fresh leaf tissue was homogenized in an ice bath with 2 mL of phosphate sodium buffer (100 mM, pH 7.5), and the homogenate was centrifuged at 8000 rpm for 15 min. Then, 0.5 mL of the supernatant was added to 5 mL of the prepared reagent (50 mg of CuSO_4_·5H_2_Owas added to 10 mL of 2% sodium tartarate; 1 mL of this solution was added to 50 mL of 2% sodium carbonate prepared in 0.1N NaOH), thoroughly mixed, and 0.5 mL of Folin–Ciocalteau reagent was added. After incubating, protein content was determined photometrically (Spectrophotometer JENWAY 6100, Dunmow, Essex, UK) using a standard curve of Bovine Serum Albumin (BSA).

### 4.4. Statistical Analysis

The experiment was arranged in a randomized complete block design (RCBD) with three replicates. Prior to analysis, data were tested for normality using the Shapiro–Wilk test. Combined analyses of variance (ANOVA) were performed to compare mean values for the different factors analyzed, using a significance level of *p* < 0.05. When significant differences were detected, means were compared using the Duncan test. Principal Component Analysis (PCA) was conducted to identify relationships among physiological and biochemical parameters. All statistical analyses were performed using Statgraphics Centurion 19 (StatPoint Technologies Inc., Warrenton, VA, USA).

## 5. Conclusions

This study highlights the remarkable adaptive diversity of traditional Moroccan fig varieties, which exhibit distinct strategies for coping with Mediterranean environmental stressors. The separation between stress-avoidance types (Nabout, Ounq Hmam) and stress-tolerant types (Ghouddane, Fassi) underscores the evolutionary significance of these local genotypes and their potential value for climate-resilient agriculture.

Traditional Moroccan fig varieties illustrate how temporal specialization can balance the trade-off between productivity and survival: some varieties capitalize on favorable conditions to maximize yield, while others maintain performance under environmental stress. This diversity of adaptive strategies functions as a natural buffer against climate variability and may serve as a vital foundation for resilient and sustainable fig cultivation.

Varietal differences reflect co-evolved adaptations rather than simple optimization traits, underscoring the importance of carefully matching varieties to local environmental conditions and anticipated climate shifts. These findings can guide growers and breeders in selecting and cultivating varieties that are better adapted to specific agroclimatic conditions.

## Figures and Tables

**Table 1:** Analyses of variance for SPAD index, total chlorophyll content (ChlT), chlorophyll fluorescence (F_v_/F_m_), canopy temperature depression (CTD), proline content, protein content, total soluble sugar (TSS), hydrogen peroxide (H_2_O_2_), and malondialdehyde (MDA) in leaves of four fig varieties grown at three locations in northern Morocco.

Variation	Df	SPAD	ChlT	F_V_/F_m_	CTD	Proline	Protein	TSS	H_2_O_2_	MDA
Location	2	13.264 ***	0.614 ***	0.00751 ***	18.0123 ***	70.926 ***	1.4238 ***	5.137 ***	1.293 ***	0.0398 ***
Variety	3	47.284 ***	0.718 ***	0.02358 ***	21.0292 ***	85.12 ***	0.4952 ***	1.2196 ***	12.764 ***	3.2001 ***
Replicate	2	0.285	0.018	0.00005	0.0003	0.929	0.0011	0.004	0.005	0.0008
Location * Variety	6	22.850 ***	0.239 ***	0.00366 ***	8.1465 ***	19.706 ***	1.3355 ***	0.469 ***	0.208 ***	0.1545 ***
Residual	22	0.187	0.010	0.00022	0.0006	1.457	0.0009	0.004	0.004	0.0007
Total (corrected)	35									

* Significant at 0.05 probability level; *** Significant at 0.001 probability level.

**Table 2:** Correlations between SPAD index, total chlorophyll content (ChlT), chlorophyll fluorescence (F_v_/F_m_), canopy temperature depression (CTD), proline content, protein content, total soluble sugar (TSS), hydrogen peroxide (H_2_O_2_), and malondialdehyde (MDA) in leaves of four fig varieties grown at three locations in northern Morocco.

	SPAD	ChlT	F_v_/F_m_	CTD	Protein	TSS	Proline	H_2_O_2_	MDA
SPAD		0.834 ***	0.299	0.216	0.186	−0.051	−0.433 **	−0.543 ***	−0.502 ***
ChlT			0.356 *	0.153	0.188	−0.229	−0.552 ***	−0.556 ***	−0.570 ***
F_v_/F_m_				0.652 ***	−0.223	−0.499 ***	−0.456 **	−0.732 ***	−0.761 ***
CTD					−0.160	−0.263	−0.295	−0.633 ***	−0.509 ***
Protein						0.556 ***	0.080	0.351 *	0.426 *
TSS							−0.113	0.580 ***	0.488 ***
Proline								0.492 ***	0.683 ***
H_2_O_2_									0.930 ***
MDA									

* Significant at 0.05 probability level; ** Significant at 0.01 probability level; *** Significant at 0.001 probability level.

## Data Availability

Data are contained within the article.
